# Effect of Surface Anesthetics on Tongue Sensory Function

**DOI:** 10.7759/cureus.101570

**Published:** 2026-01-14

**Authors:** Mami Takemori, Mika Honda-Sakaki, Wakana Nakamoto, Chieko Taguchi, Michiharu Shimosaka, Takashi Iida, Osamu Komiyama, Hidenori Yamaguchi

**Affiliations:** 1 Department of Anesthesiology, Nihon University School of Dentistry at Matsudo, Chiba, JPN; 2 Department of Anatomy, Nihon University School of Dentistry at Matsudo, Chiba, JPN; 3 Department of Anesthesiology, Nihon University Graduate School of Dentistry at Matsudo, Chiba, JPN; 4 Department of Community Oral Health, Nihon University School of Dentistry at Matsudo, Chiba, JPN; 5 Department of Prosthodontics and Oral Rehabilitation, Nihon University School of Dentistry at Matsudo, Chiba, JPN

**Keywords:** benzocaine, burning mouth syndrome (bms), lidocaine hydrochloride, mechanical detection threshold, mechanical pain threshold, numerical rating scale, quantitative sensory testing, tongue

## Abstract

Background: Lidocaine hydrochloride (LDCA) is one of the medications used to treat burning mouth syndrome (BMS). The purpose of this study is to investigate whether benzocaine (BEN) can also be used for the treatment and examination of BMS. This study used quantitative sensory testing to investigate the effects of two surface anesthetics on the sensory function of the tongue tip.

Methods: Thirty healthy women participated in this study. All participants completed a single-blind, randomized crossover study. Surface anesthetics- (2%LDCA, 20%BEN) and vaseline (control) were applied to the tongue tip. The experiment was conducted over three days, with each drug applied on a different day in a randomized order. The mechanical detection threshold (MDT), mechanical pain threshold (MPT), and numerical rating scale (NRS) were measured at the following points: before application of the drug (pre), immediately, at 5, 15, 30, and 60 minutes after application of the drug.

Results: MDT immediately (P < 0.01), at 5 (P < 0.01) and 15 (2%LDCA: P = 0.0112, 20%BEN: P = 0.0128) minutes after application of the drug were significantly higher than pre-values in both local anesthetic sessions. MPT immediately (P < 0.01), at 5 (P < 0.01), 15 (P < 0.01), and 30 (P = 0.0026) minutes after application of 2%LDCA was significantly higher than pre-values. MPT immediately (P < 0.01), at 5 (P < 0.01) and 15 (P = 0.0057) minutes after application of 20%BEN was significantly higher than pre-values. NRS scores did not differ significantly between measurement periods or between drugs.

Conclusions: This study suggested that 2%LDCA and 20%BEN may have comparable anesthetic effects on the sensory function of the healthy tongue tip.

## Introduction

Burning mouth syndrome (BMS) is a chronic pain disorder that is characterized by a burning or tingling sensation in the oral mucosa, without any clinically apparent mucosal manifestations [1,2]. BMS, which has a reported prevalence of 0.7-15% [1,3], is more common in women (especially postmenopausal women) than in men [1-3]. Though the tongue tip is the site with a predilection for BMS, the lingual border, lips, and hard and soft palates are frequently involved [2]. However, the exact pathophysiology of primary BMS remains unclear. Though it is important to identify the possible causative factors, BMS is idiopathic orofacial pain, and pharmacotherapy, in addition to behavioural therapy and psychotherapy, may help to facilitate symptom alleviation [2]. Despite the availability of many new formulations of intraoral surface anesthetics, lidocaine hydrochloride (LDCA) and benzocaine (BEN) remain the most frequently used surface anesthetics [4,5]. These anesthetics exert their effects by inhibiting sensory nerve fibre signals and are effective at a depth of 2-3 mm within or just below the mucosa [4-7], thereby effectively minimise pain during needle insertion [5]. LDCA-based surface anesthetic was sometimes used as one of the treatments for BMS and other oral pain disorders [8]. Okayasu et al. used topical LDCA as the initial treatment in patients with persistent idiopathic dentoalveolar pain, postherpetic neuralgia, and trigeminal neuralgia (TN) as well as BMS [8], and expert opinions have upheld the usefulness of this treatment strategy [9,10]. The analgesic efficacy of 8%LDCA spray for BMS was reported to be 85.6% [10]. BEN has not been studied in the treatment of BMS patients with LDCA allergy, despite its potential as an alternative to LDCA.

Quantitative sensory testing (QST) is useful for the assessment of neuropathic pain and neurological disorders in the peripheral and central areas [11]. In dentistry, many patients complain of conditions that cause pain in the intraoral or facial area, such as BMS and TN. However, there are fewer reports on QST in the intraoral than reports pertaining to QST on the body surface, and no validated test method has been established yet [11]. Okayasu et al. used QST to investigate the effects of 2%LDCA gel on the tactile threshold (mechanical detection threshold (MDT)) and pain threshold (mechanical pain threshold (MPT)) at the tongue tip, cheeks, and palms [8]. Compared to before the LDCA application, MDT and MPT significantly increased after the LDCA application on the tongue tip, cheeks, and palms. After the LDCA application, compared to the placebo, significant differences were observed in MDT only at the tongue tip, and in MPT at the tongue tip, cheeks, and palms [8]. Furthermore, Okayasu et al. used QST to study the effect of 8% LDCA spray for MDT and MPT on cheeks and palms [12]. Compared with those before LDCA application, the MDT and MPT on the cheeks and palms significantly increased after LDCA application. After the LDCA application, a significant difference was observed in the MPT on the cheeks and palms compared to the placebo, which indicated the effect of LDCA [12]. Takemori et al. previously applied 2%LDCA, 20%BEN, and vaseline as a control to the capsaicin-induced tongue pain model and examined changes in numerical rating scale (NRS) scores, MDT, and MPT [13]. The NRS scores in the capsaicin-induced tongue pain model showed similar changes among 2%LDCA, 20%BEN, and the control [13]. In the capsaicin-induced tongue pain model, the MDT showed a significant increase immediately after application of the drug compared to the control in both local anesthetic sessions [13]. In the capsaicin-induced tongue pain model, the MPT after application of 2%LDCA showed a significant increase immediately after application compared to the 20%BEN session [13]. Therefore, in the capsaicin-induced tongue pain model, the MPT differed between 2%LDCA and 20%BEN sessions.

This study used QST to investigate the effects of two types of topical anesthetics on sensory function at the tongue tip, a common site for BMS. This research may contribute to establishing diagnostic and treatment methods for BMS. Furthermore, this study can verify whether the difference in MPT between 2%LDCA and 20%BEN reported by Takemori et al. [13] is attributable to the capsaicin-induced tongue pain model or whether similar results occur in healthy tongues.

## Materials and methods

Sample size analysis

Prior to the experiment, the appropriate sample size was determined with EZR (Saitama Medical Center, Jichi Medical University, Saitama, Japan) to determine the significant difference in pain thresholds estimated using Semmes-Weinstein monofilaments (Premier Products, Kent, WA, USA). The mean and standard deviation (SD) values for pain thresholds with topical lidocaine were 2.10 ± 0.63 (Log Force) in the Okayasu et al. study [8]. Based on the performance and characteristics of repeated measurements of pain thresholds and multiple comparisons between groups, the power to detect a difference in pain threshold of 0.4 (Log Force) with a sample size of thirty participants was estimated to be 80%.

Participants

Participants comprised thirty healthy women (29 ± 4 years, range 24-39). All participants excluded individuals who had orofacial pain or temporomandibular disorders, were pregnant women, with severe mental illness, with allergic to the drug used, were scheduled for dental treatment during the measurement period, and were taking medications (antidepressants, analgesics, and sleeping pills) before 48 hours of measurements [14,15]. The Ethical Review Committee of the Nihon University School of Dentistry, Matsudo, Japan, authorized the study (no. EC24-23-025-1). Informed consent was obtained from all participants.

Study protocol

**Figure 1:**
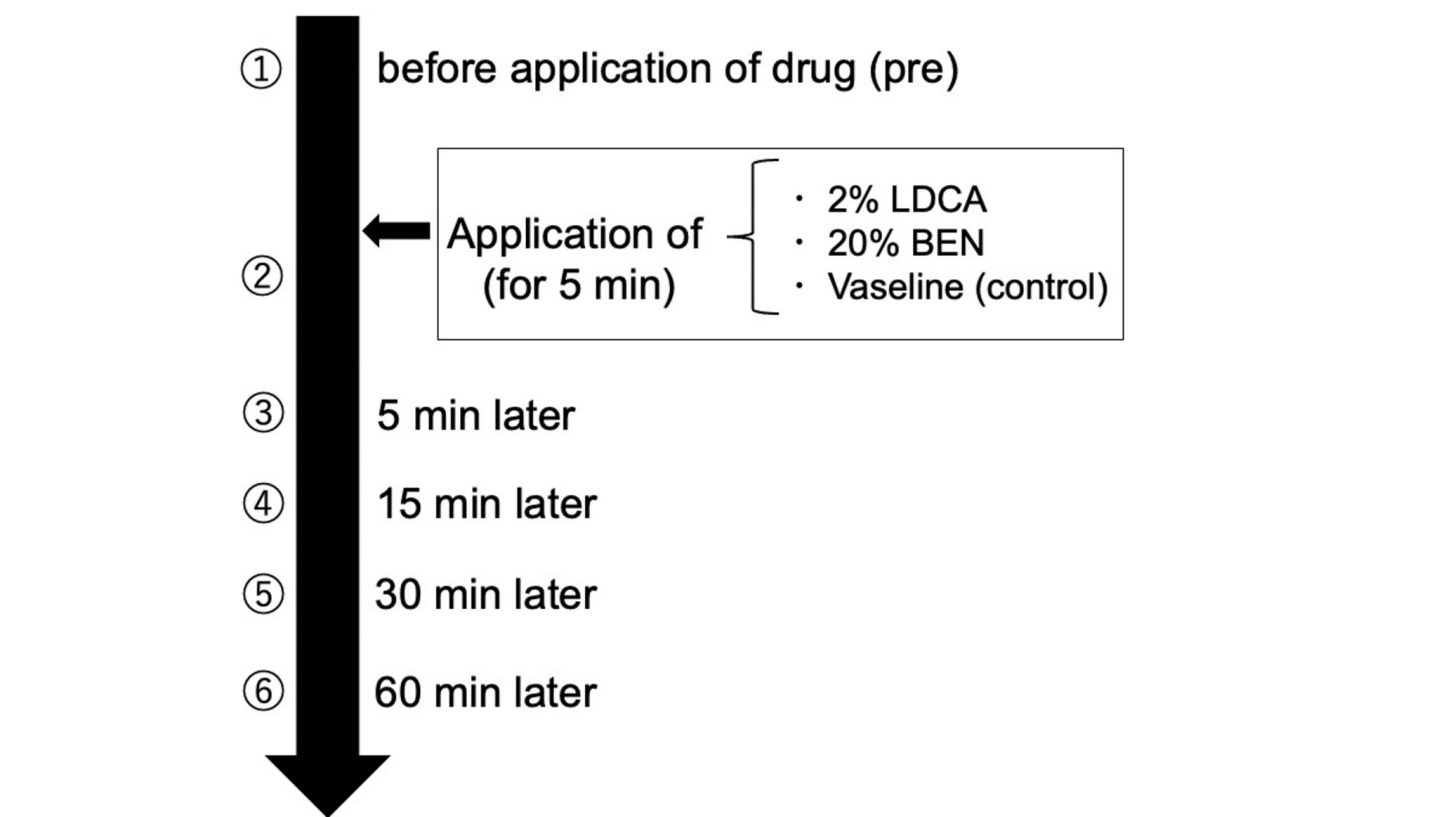
Study protocol Measurements were taken before application of the drug (pre, immediately and 5, 15, 30, and 60 minutes after application of the drug. Each participant undertook three sessions (2% lidocaine, 20% benzocaine or Vaseline) at a 3-5 days interval in randomized order. The three drugs were applied in random order. Three measurements (mechanical detection threshold, mechanical pain threshold and numerical rating scale) were measured. LDCA: Lidocaine; BEN: Benzocaine; control: Vaseline

The participants sat on the dental chair. All participants received each of the three types of drug application in a randomized crossover design. Vaseline (White Vaseline; Ono Pharmaceutical Co., Ltd., Osaka, Japan) was applied as the control. 2%LDCA gel (Xylocaine Jelly 2%; Sandoz Co., Ltd., Tokyo, Japan) or 20%BEN gel (Benzocaine dental jelly 20%, B-Brand Medico-Dental Co., Osaka, Japan) was applied as surface anesthetics. For each session, 0.2mL of each drug was dispensed into a disposable dappen glass, and the tongue tip (approximately 1 cm^2^) was immersed in the drug for 5 minutes. Measurements were taken before application of the drug (pre), immediately, at 5, 15, 30, and 60 minutes after application of the drug. A single dentist conducted all measurements. Each participant was tested in three sessions, with an interval of 3-5 days between sessions, during which a different drug application was administered in randomized order (Figure 1). All participants completed a single-blind, randomized crossover study. Three sensory function measurement methods were used: MDT, MPT, and NRS scores.

**Figure 2:**
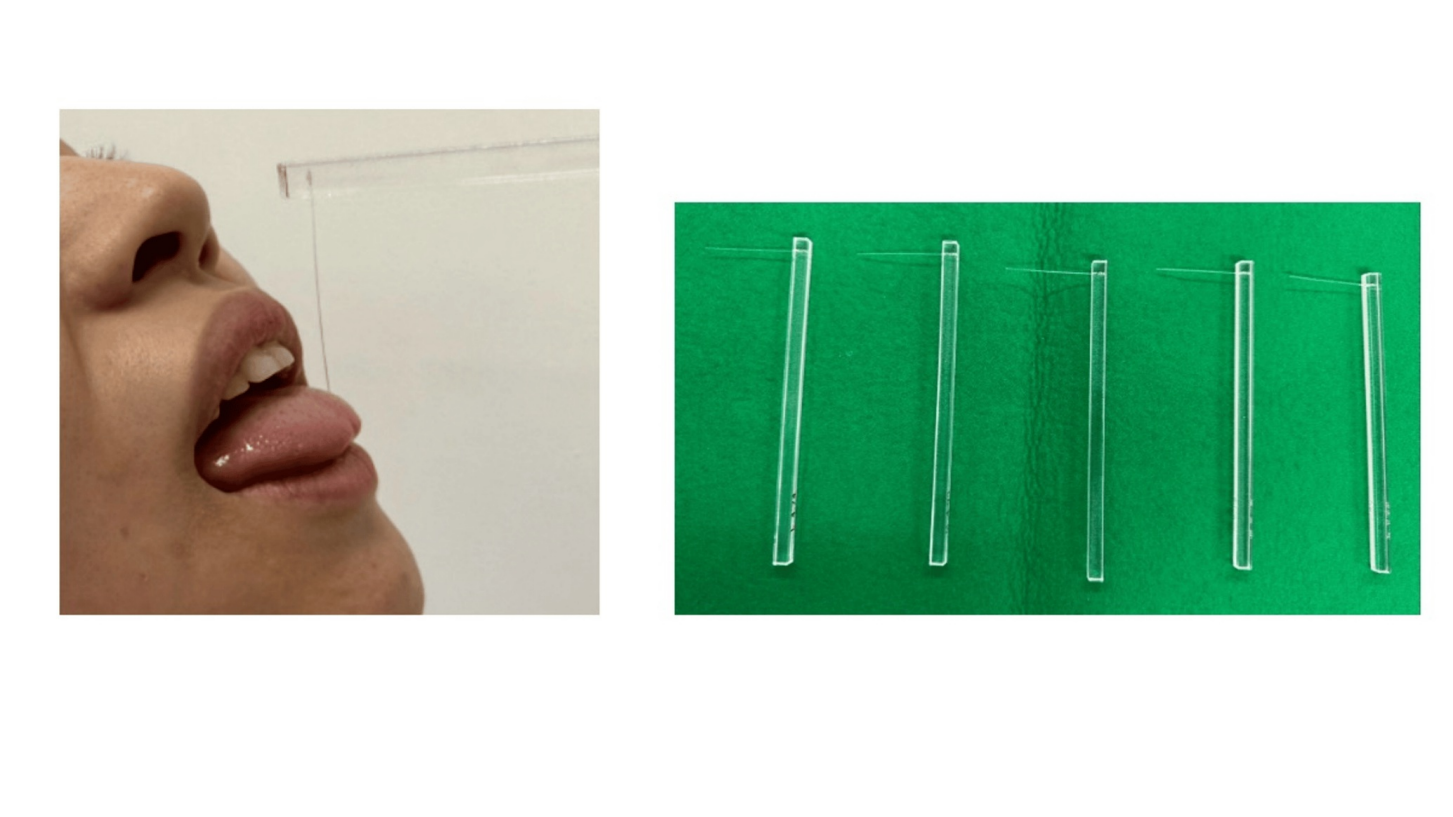
Mechanical detection threshold (MDT) measurement method Left: Measuring MDT using Semmes-Weinstein monofilament (1.5 seconds to make contact, 2 seconds to hold, and 1.5 seconds to release); Right: Semmes-Weinstein monofilament (Premier Products, Kent, WA, USA).

MDT was measured using Semmes-Weinstein monofilaments, which exert forces between 1.65 mN and 6.65 mN (Figure 2) [8,11-15]. The filament was positioned perpendicular to the tongue tip, and force was slowly applied until the filament bent. The filament was held for approximately 1.5 seconds. Measurement began with the smallest value of the Semmes-Weinstein monofilament (1.65 mN). All participants were instructed to keep their eyes closed during the MDT test and to raise their hand when they felt a sensation on the tongue tip. In case participants did not raise their hands, the response was considered a negative, and the next thicker filament was used. The thickness of the filament was increased until participants raised their hands, which was considered a positive response. The thickness was then lowered to the thinnest filament at which the participants stopped raising their hands. In case participants did not raise their hands even using the thinnest filament (1.65 mN), the thickness was halved (0.825 mN). The upper and lower limits were each measured five times, and the average values were calculated [8,11-15].

**Figure 3:**
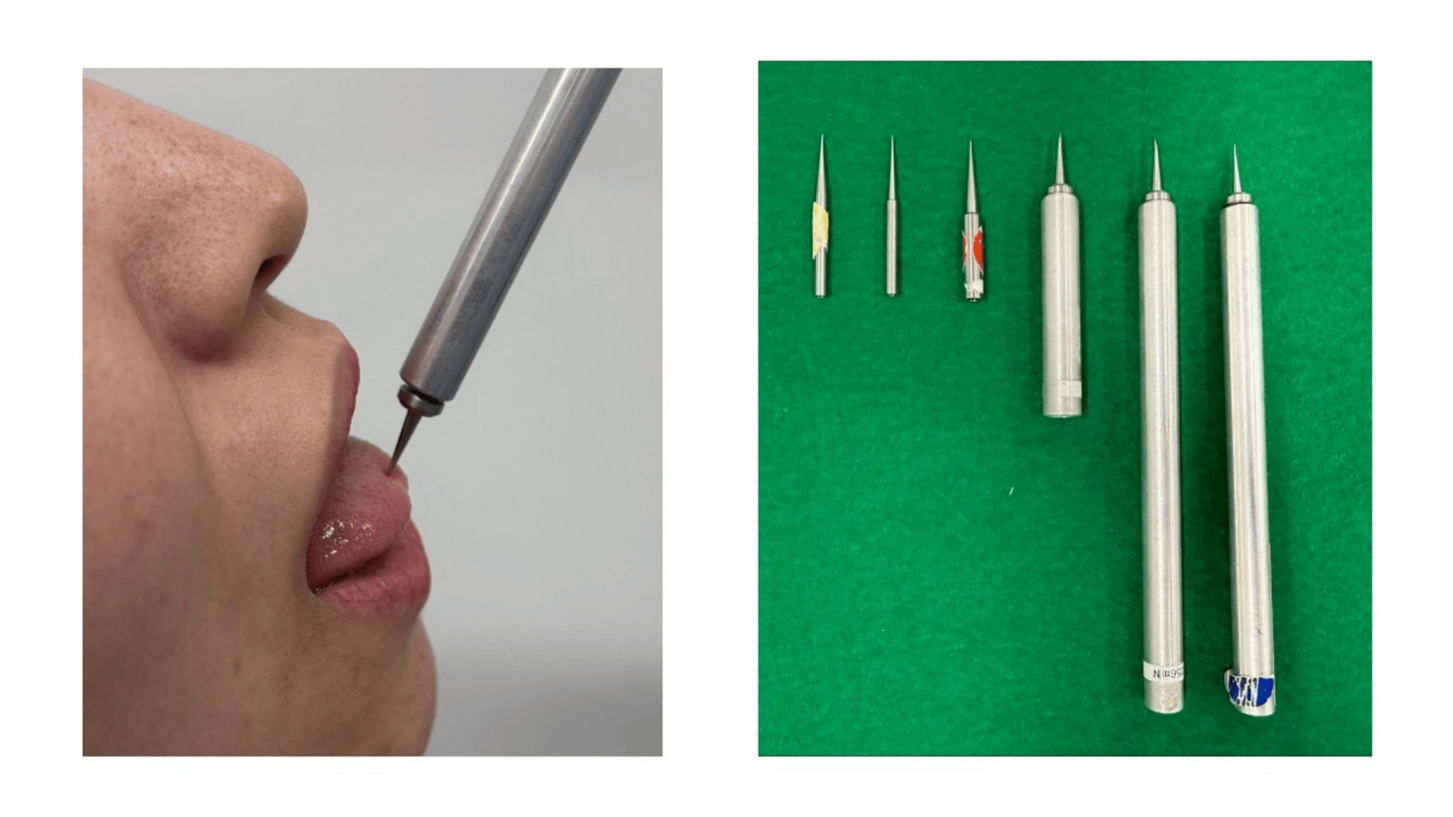
Mechanical pain threshold (MPT) measurement method Left: Measuring MPT using a pinprick stimulator (Intercross Co., Ltd., Tokyo, Japan); Right: Pinprick stimulator.

After MDT measurements, MPT was examined in the same manner. MPT was measured using a pinprick stimulator (Intercross Co., Ltd., Tokyo, Japan) with fixed stimulus intensities between 8 mN and 512 mN (Figure 3) [8,11-15]. The tongue tip was stimulated for approximately 2 seconds. Measurements were obtained, starting with the smallest value obtained using a pinprick stimulator (16 mN). All participants were instructed to close their eyes during the test and raise their hands when they experienced pain at the tongue tip. In case participants did not raise their hands, the response was considered a negative, and the next heaviest pinprick stimulator was used. The weight was increased until participants raised their hands, which was considered a positive response. In case participants did not raise their hands even using the heaviest pinprick stimulator (512 mN), the weight was doubled (1024 mN). Measurements were performed in ascending or descending order of stimulus intensity, starting with the pinprick device delivering the lowest value. The upper and lower limits were each measured five times, and the average values were calculated [8,11-15].

Pain intensity of MPT was assessed on the NRS score. NRS score was measured as a subjective rating on a scale from 0 to 10 for pinprick stimulation pain (0 = no pain, 10 = maximum imaginable pain) [8,11-15]. The study period was from November 25, 2024, to March 29, 2025.

Statistical analysis

The normality of data was tested with the Kolmogorov-Smirnov test. As normality was not observed for MDT, MPT, and NRS scores, the Friedman test was used to evaluate the differences in the time course of each drug. The Kruskal-Wallis test was used to evaluate the differences between the drugs at each measurement time. If the Kruskal-Wallis test revealed a significant difference, a post-hoc analysis was performed using the Steel-Dwass test. Excel-Toukei Ver 4.06 (for Windows; Bell Curve for Excel, SSRI Co., Ltd., Tokyo, Japan) was used for these analyses, and a P-value of <0.05 was considered to indicate statistical significance.

## Results

Table 1 shows MDT, MPT and NRS scores over time, are summarized with the corresponding P-values.

**Table 1:** Mechanical detection threshold, mechanical pain threshold and numerical rating scale of 2% lidocaine, 20% benzocaine and Vaseline sessions at the measurement time Data are expressed as mean (standard error of the mean). MDT: Mechanical detection threshold, MPT: Mechanical pain threshold, NRS: Numerical rating scale. LDCA: Lidocaine, BEN: Benzocaine, control: Vaseline *P < 0.05, **P < 0.01 vs control.

Measurement	Session	Time					
		Pre	Immediately	5 min	15 min	30 min	60 min
MDT [mN]	2%LDCA	1.26 (0.03)	2.26 (0.12)**	2.28 (0.15)**	1.78 (0.13)**	1.5 (0.09)	1.32 (0.05)
	20%BEN	1.22 (0.02)	2.35 (0.09)**	2.07 (0.10)**	1.62 (0.09)*	1.42 (0.06)	1.34 (0.06)
	control	1.24 (0.02)	1.39 (0.07)	1.35 (0.06)	1.31 (0.05)	1.36 (0.05)	1.32 (0.05)
MPT [mN]	2%LDCA	108.41 (17.82)	230.76 (29.85)**	208.24 (29.16)**	193.19 (30.68)	173.18 (27.89)	143.52 (23.43)
	20%BEN	105.46 (14.25)	246.32 (33.65)**	210.76 (28.83)**	179.57 (26.83)	167.32 (27.30)	157.71 (27.08)
	control	102.60 (16.80)	115.80 (16.74)	114.29 (16.96)	112.53 (12.41)	111.80 (12.98)	107.92 (16.11)
NRS	2%LDCA	2.74 (0.19)	2.29 (0.22)	2.16 (0.23)	2.35 (0.25)	2.52 (0.23)	2.58 (0.23)
	20%BEN	2.74 (0.20)	2.23 (0.25)	2.13 (0.24)	2.39 (0.23)	2.42 (0.26)	2.42 (0.24)
	control	2.71 (0.21)	2.68 (0.24)	2.71 (0.26)	2.71 (0.24)	2.71 (0.24)	2.74 (0.25)
* P < 0.05, ** P < 0.01							

**Figure 4:**
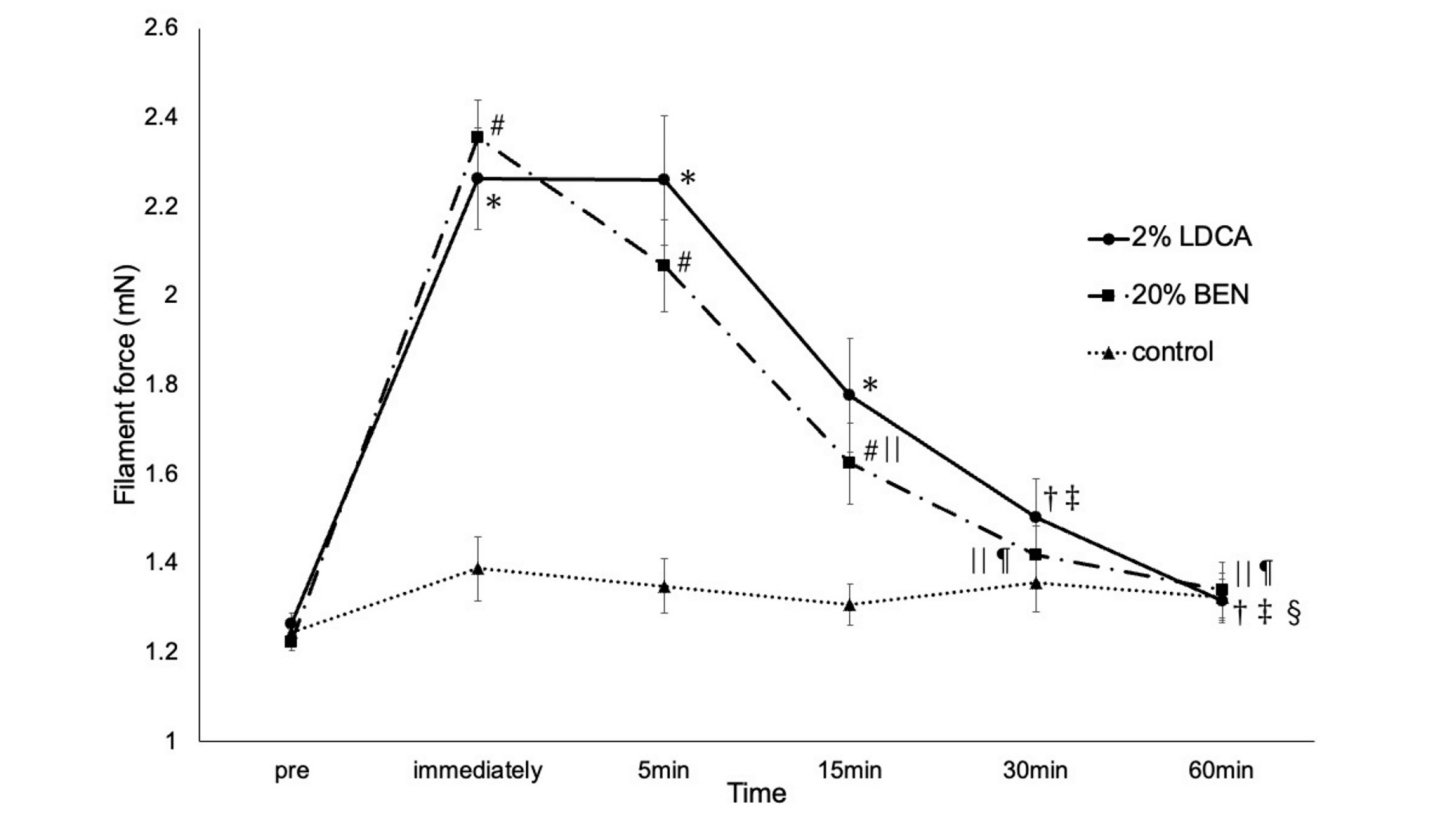
Mechanical detection threshold for each agent Data are shown as means; bars are standard error. *: P < 0.01 when compared to pre in the 2%LDCA session. †: P < 0.01 when compared to immediately after application of the drug in the 2%LDCA session. ‡: P < 0.01 when compared to 5 minutes after application of the drug in the 2%LDCA session. §: P < 0.05 when compared to 15 minutes after application of the drug in the 2%LDCA session. #: P < 0.01 when compared to pre in the 20%BEN session. ||: P < 0.01 when compared to immediately after application of the drug in the 20%BEN session. ¶: P < 0.01 when compared to 5 minutes after application of the drug in the 20%BEN session. LDCA: Lidocaine; BEN: Benzocaine; control: Vaseline

Figure 4 shows the time course of MDT changes after application of 2%LDCA, 20%BEN and control. Before the application of the drug, MDT was comparable among the three sessions. Immediately after drug application, MDT in both local anesthetic sessions was significantly higher than pre-value (P < 0.01). At 5 minutes after application of the drug, MDT in both local anesthetic sessions was significantly higher than the pre-value (P < 0.01). At 15 (2%LDCA : P = 0.0112, 20%BEN : P = 0.0128) minutes after application of the drug, MDT in both local anesthetic sessions was significantly higher than the pre-value. In the 2%LDCA and control sessions, a significant difference was found immediately (P < 0.01), at 5 (P < 0.01) and 15 (P = 0.0026) minutes after application of the drug. In the 20%BEN and control sessions, a significant difference was found immediately (P < 0.01), at 5 (P < 0.01) and 15 (P = 0.0141) minutes after application of the drug.

**Figure 5:**
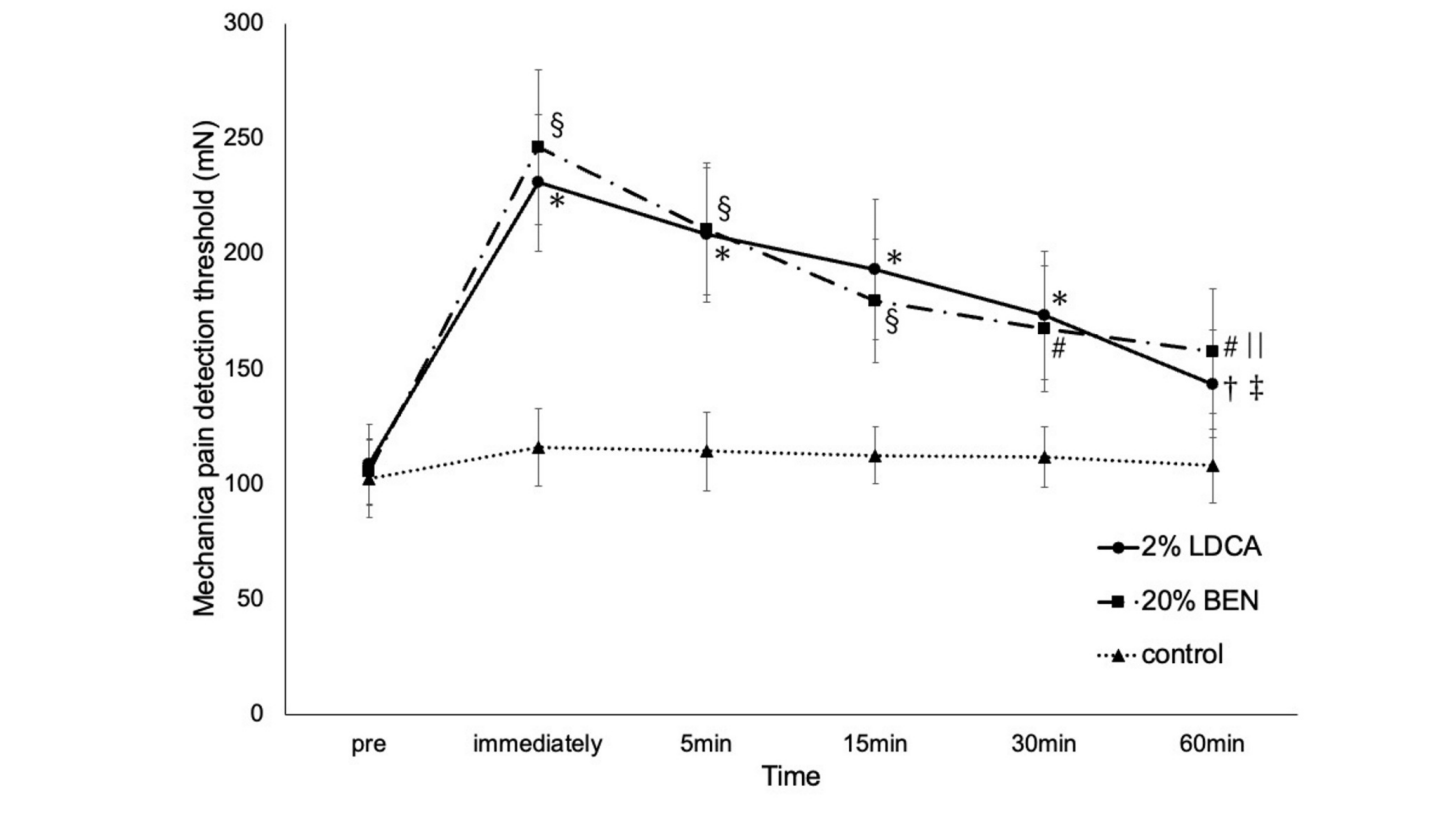
Mechanical pain threshold for each agent Data are shown as means; bars are standard error. *: P < 0.01 when compared to pre in the 2%LDCA session. †: P < 0.01 when compared to immediately after application of the drug in the 2%LDCA session. ‡: P < 0.01 when compared to 5 minutes after application of the drug in the 2%LDCA session. §: P < 0.01 when compared to pre in the 20%BEN session. #: P < 0.01 when compared to immediately after application of the drug in the 20%BEN session. ||: P < 0.01 when compared to 5 minutes after application of the drug in the 20%BEN session. LDCA: Lidocaine; BEN: Benzocaine; control: Vaseline

Figure 5 shows the time of MPT changes application of 2%LDCA, 20%BEN and control. Before the application of the drug, MPT was comparable among the three sessions. Immediately after drug application, MPT in both local anesthetic sessions was significantly higher than the pre-value (P < 0.01). At 5 minutes after application of the drug, MPT in both local anesthetic sessions was significantly higher than the pre-value (P < 0.01). At 15 minutes after application of the drug, MPT in the 20%BEN session was significantly higher than the pre-value (P = 0.0057). At 15 (P < 0.01) and 30 (P = 0.0026) minutes after application of the drug, MPT in the 2%LDCA session was significantly higher than the pre-value. By 60 minutes after application of the drug, MPT in both local anesthetic sessions continued to decline and approached pre-value. In both local anesthetic and control sessions, a significant difference was found immediately (2%LDCA : P = 0.0013, 20%BEN : P = 0.0036) and at 5 (2%LDCA : P = 0.0049, 20%BEN : P = 0.0073) minutes after application of the drug.

**Figure 6:**
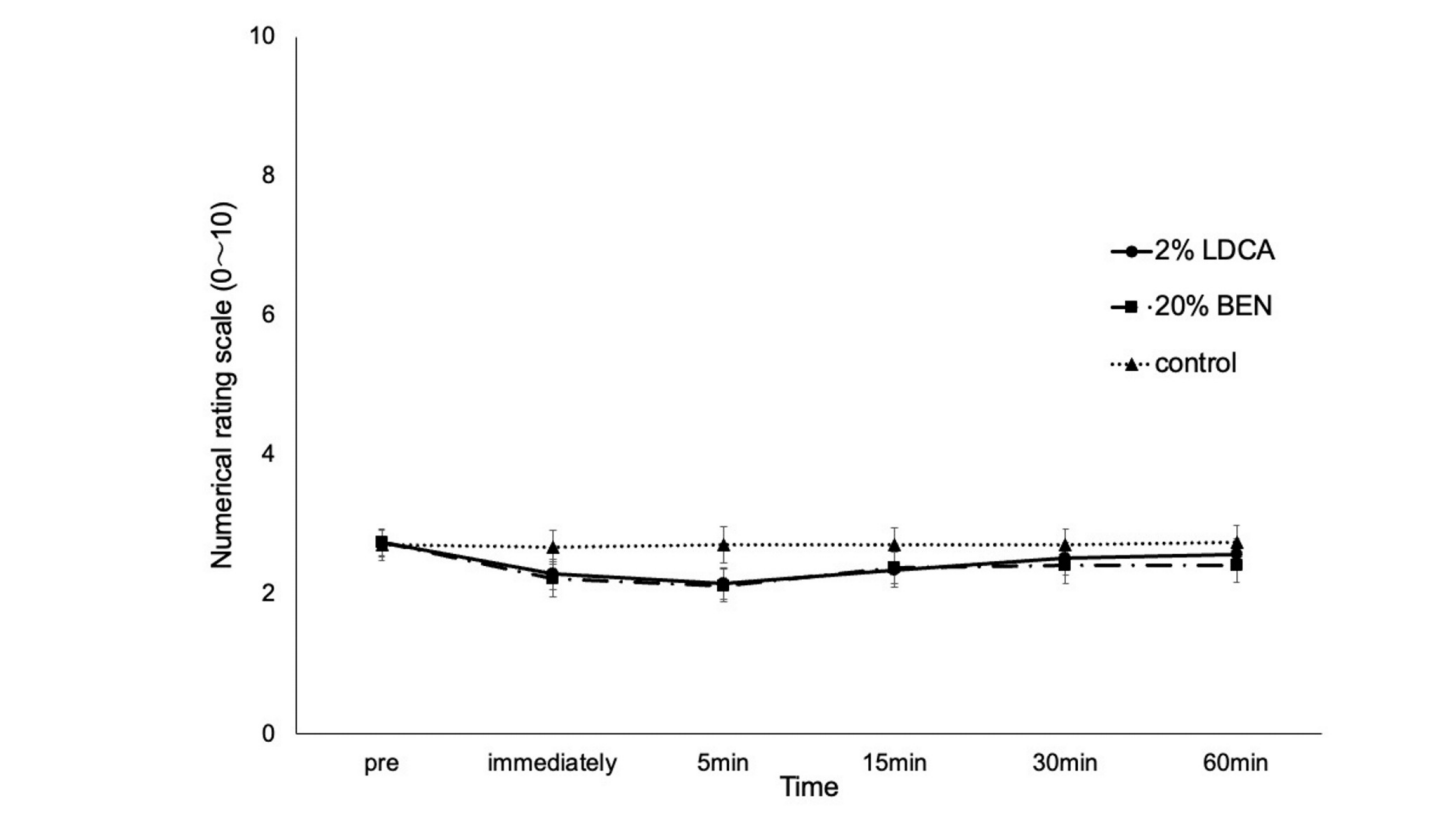
Numerical rating scale for each agent. Ratings on a 0-10 scale. Data are shown as means; bars are standard error. No significant differences between sessions. LDCA: Lidocaine; BEN: Benzocaine; control: Vaseline

Figure 6 shows the time course of NRS score changes after the application of 2%LDCA, 20%BEN and control. The NRS scores did not differ significantly between measurement periods or between sessions.

## Discussion

The major local anesthetics currently used in clinical dental practice can be classified into two categories: amide-linked anesthetics, such as LDCA and dibucaine, and ester-linked anesthetics, such as BEN [6,7]. LDCA is the most widely used infiltration anesthetic [5,7]. It acts by blocking sodium channels, and the same local administration blocks ectopic discharges from afferent fibres, such that local administration slows down the sensitization of peripheral nociceptors and increases central excitability [16]. It has strong tissue permeability and excellent anesthetic effects. The lower water solubility of BEN, an ethyl para-aminobenzoate, allows for its rapid absorption into mucous membranes and prolongs its action [5]. Several local allergic reactions have been reported, and methemoglobinemia has been reported to occur when it is used in large doses [17,18]. However, this is rare in the literature and with the amount used in dentistry [5]. A wide variety of surface anesthetics are available, including gels, sprays, ointments, and adhesive patches [6]. Gel surface anesthetics are commonly used in various patients (including children) and procedures to relieve pain during infiltration anesthesia needle punctures. This study used LDCA gel and BEN gel to avoid differences due to the dosage forms of the drugs. Park et al. reported that the onset time of 20%BEN is 1 minute compared with 3 minutes for 10% LDCA [19]. Lee et al. reported that the onset time of BEN is 1 minute and the duration of 10 minutes, compared with the onset time of LDCA is 2 minutes and the duration of 15 minutes [7]. Smrkolj et al. reported that the onset time of LDCA is 167 seconds, and the duration of the action half-life is 41 minutes [20]. The studies employing surface anesthetic application durations of 1-3 minutes have reported mixed results [21,22]. Alqareer et al. applied 20%BEN to the maxillary canine attachment gingiva for 5 minutes and assessed the pain caused by needle puncture using the visual analogue scale (VAS) [4]. BEN had lower VAS than placebo [4]. Therefore, in this study, the drug application time was set at 5 minutes, and the measurement time was limited to 60 minutes. This is the time during which both local anesthetics are reliably effective. Rosa et al. applied 5% LDCA gel and 20%BEN gel to the palatal mucosa and evaluated the pain caused by needle puncture using the VAS [23]. The VAS was lower for LDCA and BEN than for placebo [23]. Rosivack et al. applied 5% LDCA gel and 20%BEN gel to the adherent gingiva of the maxillary canine teeth and evaluated the pain caused by needle puncture using the VAS and measuring the heart rate of patients [24]. The VAS was lower for LDCA and BEN than for placebo, and the heart rate did not differ significantly between the three drugs [24]. In a study conducted in children, Ponniah et al. applied 2%LDCA gel and 20%BEN gel to the oral mucosa and evaluated needle puncture pain using a 4-point scale [6]. 20%BEN gel was more effective than 2%LDCA gel [6]. The studies comparing LDCA and BEN have reported on the effects on acute pain, but no comparative studies have reported on the effects on chronic pain, such as BMS. Several studies have found that pain measurements assessed using self-rating scales varied with age and sex [25]. Women tend to become less sensitive to pain with age [25]. Moreover, women are more sensitive to pain than men and often have lower pain thresholds [26]. Therefore, the present study was limited to healthy women aged 20-30 years. Clinically, a difference of at least 3 points in the NRS scores was considered significant [27,28]. Kanai et al. and Niki et al. reported that the application of 8% LDCA spray to the oral mucosa significantly reduced NRS scores from 5 to 1 in patients with TN and from 6 to 1 in patients with post-herpetic neuralgia [27,28]. In this study, all NRS scores at the time of MPT measurement at all time points were below 3 points. In this study, no significant differences were observed in the NRS scores at any measurement time compared to before application of the drug in healthy tongues. NRS scores may be a valid measure of neuropathic pain and other types of pain [11]. The VAS, 4-point scale, and the NRS scores are all self-rating scales, and significant variability may occur in individual subjective responses [15]. Although many reports on the effects of local anesthetics use self-rating scales, investigating changes in somatic sensation is necessary to elucidate the somatosensory afferent nervous system and pathophysiological mechanisms underlying pain occurrence [15].

QST is a useful tool to study somatosensory function and can help to study pain [14]. MDT indicates the response to Aβ fibres, and MPT indicates the response to Aδ and C fibres [29]. Subjective pain of pinprick stimulation was assessed using NRS scores. Moreover, QST is thought to help determine whether neuropathic pain is related to peripheral rather than central factors [12]. In this study, MDT was significantly elevated immediately, at 5 and 15 minutes after application of the drug, and MPT was significantly elevated immediately and 5 minutes after application of 2%LDCA and 20%BEN compared to the control. This is consistent with the report by Okayasu et al. [8], who applied 2%LDCA gel to the tongue tip and examined the effects of MDT and MPT. MDT and MPT in this study did not differ significantly between the 2%LDCA and 20%BEN sessions at any measurement time. Therefore, the two local anesthetics were considered to have equivalent anesthetic effects. This study found that 2%LDCA and 20%BEN increased both MDT and MPT on healthy tongues. Therefore, it was demonstrated using QST that the local anesthetics of LDCA and BEN exert anesthetic effects on healthy tongues. Takemori et al. observed a significant difference in MDT but not MPT between the 20%BEN and the control in a capsaicin-induced tongue pain model [13]. The capsaicin-induced tongue pain model suggested that the anesthetic effect of 20%BEN may be reduced.

This study suggests equivalent anesthetic effects of 2%LDCA and 20%BEN on healthy tongues' sensory function. One of the limitations of this study was the drug concentrations. The 2%LDCA gel and 20%BEN gel were used in this study. However, changing the drug concentration could produce results different from those that were observed in the present study. Another limitation of this study was the sex and age of the participants. This study was conducted on the tongues of healthy women aged 20-30 years. Different results may be obtained that the tongues of healthy men or older women, the common age of patients with BMS, are considered.

BMS is classified as idiopathic orofacial pain and is further classified as (1) BMS without somatosensory changes, (2) BMS with somatosensory changes, or (3) probable BMS [30]. Category (2) is defined as "an intraoral burning or dysaesthetic sensation, recurring daily for more than 2 hours per day for more than 3 months and accompanied by negative and/or positive somatosensory changes, without evident causative lesion(s) on clinical examination and investigation [30]". This study is expected to contribute to the classification of BMS using QST in peripheral nerves. Investigating the sensory function of the tongue in normal participants will not only help in the accurate evaluation of criteria for neuropathic pain, such as BMS, but also in the selection of treatment modalities, evaluation of the healing process, and other endeavours [8,11-13]. Further research will examine changes in somatosensory responses by applying topical anesthetics to BMS, a chronic pain.

## Conclusions

In conclusion, this study investigated changes in sensory function using QST after application of 2%LDCA and 20%BEN to healthy tongues. Local anesthetics of LDCA and BEN produced similar changes in sensory function on healthy tongues. Therefore, the difference observed in MPT between 2%LDCA and 20%BEN in the capsaicin-induced tongue pain model may be attributable to the pain caused by capsaicin. The findings of this study provide a framework for future studies on the effect of 2%LDCA and 20%BEN on the sensory function in the tongue of BMS as investigated using QST.
